# Implementation of Antigen-Based Diagnostic Assays for Detection of Histoplasmosis and Cryptococcosis among Patients with Advanced HIV in Trinidad and Tobago: A Cross-Sectional Study

**DOI:** 10.3390/jof10100695

**Published:** 2024-10-05

**Authors:** Ayanna Sebro, Jonathan Edwards, Omar Sued, Leon-Omari Lavia, Tricia Elder, Robert Jeffrey Edwards, Patrick Eberechi Akpaka, Nadia Ram-Bhola, Roanna Morton-Williams Bynoe, Yanink Caro-Vega, Isshad John, Freddy Perez

**Affiliations:** 1 National AIDS Coordinating Committee, Office of the Prime Minister, Port of Spain 190126, Trinidad and Tobago; ayanna.sebro@gov.tt; 2 HIV/AIDS Coordinating Unit Ministry of Health, Port of Spain 101002, Trinidad and Tobago; jonathan.r.a.edwards@gmail.com (J.E.); omari.lavia@health.gov.tt (L.-O.L.); tricia.elder@health.gov.tt (T.E.); nadia.ram-bhola@health.gov.tt (N.R.-B.); roanna.m-w-bynoe@health.gov.tt (R.M.-W.B.); isshad.john@health.gov.tt (I.J.); 3 Department of Communicable Diseases Prevention, Control, and Elimination, Pan American Health Organization, Washington, DC 20037, USA; suedoma@paho.org; 4 Medical Research Foundation, 7 Queens Park East, Port of Spain 101002, Trinidad and Tobago; jeffreye2000@gmail.com; 5 Department of Paraclinical Sciences, The University of the West Indies, St. Augustine 330912, Trinidad and Tobago; eberechipatrick.akpaka@uwi.edu; 6 Departamento de Infectología, Instituto Nacional de Ciencias Médicas y Nutrición Salvador Zubirán, Tlalpan, México City 14080, Mexico; yanink.caro@infecto.mx; 7 Department of Community Health, Federal University of Health Sciences of Porto Alegre (UFCSPA), Porto Alegre 90050-170, RS, Brazil

**Keywords:** histoplasmosis, cryptococcosis, HIV, antigen-based diagnostic assays, Trinidad and Tobago

## Abstract

The Caribbean continues to have high HIV prevalence globally with concurrently high mortality rates due to opportunistic Infections. This study addresses the prevalence of histoplasmosis and cryptococcosis among patients living with advanced HIV disease (AHD) in Trinidad and Tobago, focusing on the implementation of antigen-based diagnostic assays. Conducted as a cross-sectional survey across five HIV treatment sites, 199 participants with advanced HIV disease were enrolled between July 2022 and September 2023. Diagnostic testing was performed using the Clarus Histoplasma Galactomannan Enzyme Immunoassay (EIA), and the Immy CrAg^®^ LFA Cryptococcal Antigen Lateral Flow Assay on urine and blood samples, respectively. Results revealed that 14.6% of participants were found to be co-infected with either histoplasmosis or cryptococcosis, with histoplasmosis being more prevalent (10.5%) than cryptococcosis (4.0%). The study found no significant demographic differences between newly diagnosed and previously diagnosed participants. However, a lower median CD4 count was associated with a higher risk of fungal opportunistic infections. The findings underscore the critical role of systematic use of fungal antigen-based diagnostic assays among patients with AHD to improve the timely diagnosis and treatment of fungal infections among people living with HIV in resource-limited settings and to improve patient outcomes and survival.

## 1. Introduction

Globally, the Joint United Nations Programme on HIV/AIDS (UNAIDS) reported that approximately 39.9 million people were living with the Human Immunodeficiency Virus (HIV) in 2023, resulting in nearly 630,000 deaths from Acquired Immune Deficiency Syndrome (AIDS)-related diseases in that same year. This represents a 52% improvement compared to 2010 [1]. However, according to UNAIDS [2,3], the Caribbean has the second highest HIV prevalence globally, with varying rates among adults across Caribbean countries ranging from approximately 1.5% in Jamaica to approximately 0.1% in Cuba [2,3]. In 2021, the estimated HIV prevalence rate among adults aged 15 to 49 in Trinidad and Tobago was 1.0% [4].

Undiagnosed, defaulting from, and/or inadequately treated HIV infection can lead to advanced HIV disease (AHD), defined by a CD4 cell count of less than 200 cells/mm^3^ or the presence of an AIDS-defining illness [5]. Individuals with low CD4 counts have an increased likelihood of contracting or re-activating a wide range of opportunistic infections (OIs) including histoplasmosis, cryptococcosis, and tuberculosis (TB) [6,7]. These OIs continue to be a major cause of death among patients with HIV in low- and middle-income countries [8,9,10]. UNAIDS estimates that between 200 and 500 adults and children succumb to AHD yearly in Trinidad and Tobago [4]. Accurate identification of disseminated OIs is challenging due to their non-specific clinical signs and symptoms, often resulting in misdiagnosis and suboptimal treatment [8]. 

In Trinidad and Tobago, the proportion of HIV-positive individuals presenting AHD ranges between 30 and 45% [11]. The first cases of HIV diagnosed in Trinidad and Tobago in 1983 were noted to involve disseminated histoplasmosis, leading to its recognition as an AIDS-defining illness [12,13]. This followed the first reported case of AIDS in an individual with cryptococcal meningitis [14]. Recent studies conducted by the Medical Research Foundation of Trinidad and Tobago, the largest HIV treatment and care site in the country, have identified cryptococcosis and histoplasmosis as significant infections, with probable incidence of disseminated disease affecting 2.5% and 6.4% of patients with AHD, respectively [15].

The systematic implementation of testing for these opportunistic fungal infections is crucial for an HIV/AIDS public health program to ensure the long-term survival of people living with HIV (PLHIV) who present advanced diseases. Various laboratory tests are used to identify the organisms responsible for cryptococcosis and histoplasmosis, including conventional gold-standard procedures such as culture, microbiological stains, and histopathology. These methods require skilled laboratory and medical personnel, laboratories with appropriate biosafety level (for culturing histoplasma), and a long turnover period for results [16,17].

Historically, Trinidad and Tobago has relied on clinical, histopathology, and antibody testing investigations for the diagnosis of these OIs, which are available in limited settings. However, these methods with low sensitivity often result in late or missed diagnoses. Alternatively, newer internationally validated rapid diagnostic assays (RDAs) and other antigen-based tests are available, capable of detecting the circulating antigens of cryptococcosis and histoplasmosis in biological specimens, with high sensitivity (>90%) and specificity (≥90%) [3,17,18,19]. These methods offer the advantage of being less invasive and can be conducted in laboratories with minimal infrastructure (biosafety levels 1 and 2) by less-specialized technicians. 

By eliminating the need for pathological and culture components in diagnosis, these methods significantly reduce the turnaround time for results, enabling healthcare providers to initiate appropriate and timely treatment [8,9,17,19,20,21,22,23]. Additionally, there is the added benefit of reduced number of patients who may be infected or co-infected with multiple OIs that are lost to follow-up. 

The objective of this study is to determine the prevalence of cryptococcosis and histoplasmosis in people living with advanced-stage HIV in Trinidad and Tobago. 

## 2. Materials and Methods

### 2.1. Study Design

We conducted a cross-sectional survey among adult PLHIV with advanced disease in Trinidad and Tobago. Surveillance was carried out across all five adult HIV treatment and care sites between July 2022 and September 2023. The overall sample size was determined to be 250 using Buderer’s formula for sensitivity, with input variables derived from the existing literature on the national prevalence of HIV [22,23,24]. The total sample size was proportionately distributed across the study locations based on the documented number of new and adult patients living with HIV who initiated antiviral therapy (ART) in the year 2020. Participants were followed up individually for 30 days post-enrolment to document treatments provided, diagnoses, and outcome status with passive follow-up during routine clinic visits for significant changes in their clinical picture. 

### 2.2. Study Implementation 

A Systems Capacity Assessment was conducted across the laboratories of the five Regional Health Authorities to evaluate the testing capacities of RDAs and conventional diagnostics, ensuring national access to tests within the public sector. The health system was also notified of the need to ensure an adequate supply of antifungal therapy, in line with treatment guidelines, due to the potential increase in diagnosed cases resulting from the study.

Laboratory technicians from all five RHAs were trained on the Immy CRAG Cryptococcal Antigen Lateral Flow Assay and the Histoplasmosis Enzyme Immunoassay (EIA) Immy test. Clinical teams were engaged prior to and during the study to support orientation to the study protocol, address study challenges, and facilitate communication for patient access to treatment. After data collection, the study coordinator and the treatment and care coordinator ensured that the results were communicated to the patients to facilitate clinical follow-up. 

### 2.3. Study Participants

Participants included hospitalized or non-hospitalized adults living with HIV with AHD (defined as a CD4 cell count less than 200 cells/mm^3^, CD4% less than 14%, or WHO clinical stage 3 or 4). Participants with previous cryptococcosis and histoplasmosis infections were excluded from the analysis.

### 2.4. Data Collection

Participants were enrolled at the various HIV treatment and care sites through purposive sampling methods. Patients meeting the inclusion criteria were recruited after obtaining informed consent. De-identified demographic information, clinical signs and symptoms, history of OIs, risk factors and potential exposures, ART status, and relevant biological markers were recorded for each participant. All information was anonymized and securely stored using the Google Form platform, which was utilized to digitize manual study forms and create a study database in an MS Excel format.

Urine samples for histoplasma antigen testing and blood samples for serum Cryptococcal Antigen testing were collected at enrolment. CD4 and viral load testing were performed if recent results (within the last three months) were unavailable. The collected urine samples were transported to the laboratory at 2–8 °C where they were frozen at −16 to −24 °C, subsequently batched, thawed to 20 to 25 °C, and processed. The testing process was completed within two weeks. Similarly, sera samples for CrAg testing were transported under similar conditions and frozen at ≤−20 °C for a maximum of one week before testing [24].

### 2.5. Laboratory Methods

Patients presenting with symptoms of late-stage disease were evaluated and tested for histoplasmosis and cryptococcosis at their initial encounter with the health system to minimize loss to follow-up. All other patients were tested for CD4 counts upon clinic entry to assess their immune status. Those meeting the study criteria based on CD4 counts were then tested for histoplasmosis and cryptococcosis during their follow-up assessment.

Histoplasmosis testing was conducted on 10 mL midstream clean catch urine samples using the Clarus Histoplasma Galactomannan EIA (HGM201) test. Cryptococcal antigen testing was performed using the Immy CrAg^®^ LFA Cryptococcal Antigen Lateral Flow Assay. For this test, 10 mL of whole blood was collected in clot-activator tubes and subsequently centrifuged to obtain serum. Histoplasmosis testing was conducted at the Medical Research Foundation of Trinidad and Tobago (MRFTT) and North Central Regional Health Authority’s laboratories. Concurrently, cryptococcal testing took place at the national laboratories at MRFTT, North Central Regional Health Authority (NCRHA), Eastern Regional Health Authority, Southwest Regional Health Authority, and Tobago Regional Health Authority laboratories. Whole blood samples for CD4 testing were sent to the MRFTT’s laboratory, where testing was performed using the Becton Dickson FACS Presto™ CD4 cartridges. Viral load testing was conducted using the Abbott m2000 system. All testing was integrated within a networked system, ensuring that tests were available at all facilities and promptly reported to the clinical team to support appropriate management and treatment. 

### 2.6. Data Cleaning and Statistical Analysis

Data were transferred from Google Form to an Excel file, where they were reviewed, cleaned, and corrected. Responses for study variables were checked for erroneous formats, and inconsistent, incomplete, or missing data were monitored and addressed. Outlier results were re-examined to verify the accuracy of reporting. A 30-day follow-up was conducted to determine and classify outcome status as dead, alive, or lost to follow-up. Duplicate respondents within the database were identified and filtered out, and the completeness of respondent data was ensured by combining information from both entries. 

#### 2.6.1. Statistical Analysis

We analyzed sociodemographic and clinical characteristics, including sex, current age, employment, new ART usage, CD4 cell count, patient type (hospitalized or outpatient), and diagnoses of fungal opportunistic infections during follow-up. Participants were classified as having a “new HIV diagnosis” if their diagnosis date was within 180 days of study enrolment; those diagnosed more than 180 before enrolment were categorized as having a “previous HIV diagnosis”. New ART was defined for participants whose ART occurred between 30 days before and 30 days after the study entry. Frequencies and percentages were used to describe the characteristics, while chi-square tests and Kruskal–Wallis tests were employed to compare groups. We also described the number of participants with fungal opportunistic infections by CD4 cell count, evaluated the presence of risk factors for cryptococcosis and histoplasmosis, and reviewed specific antifungal treatment and mortality. 

We used logistic regression models on the complete dataset to explore characteristics associated with fungal opportunistic infections and applied multiple imputations with five replications to address missing CD4 count values. Splines for CD4 and age variables were used to avoid linearity assumptions. Kaplan–Meier curves were employed to explore time to death from study entry, both overall and at 30 days, with survival curves compared by fungal opportunistic infection status using the survdiff test. All analyses were performed using R Studio version 4.3.1.

#### 2.6.2. Ethical Approval 

Participation in the study was voluntary and required a signed informed consent. The study was conducted based on a protocol approved by the PAHO Ethical Committee (PAHOERC) under register PAHOERC.0347.01, the Ministry of Health of Trinidad and Tobago, and by the ethical committees of all participants, Regional Health Authorities and institutions in Trinidad and Tobago. No personally identifiable information was collected in the database used for the analysis. Data were compiled by trained professionals and stored in a secure database.

## 3. Results

### 3.1. Participant Demographics

From July 2022 to September 2023, a total of 260 individuals were initially screened, and samples were collected by participatory clinics. After reviewing CD4 results and other documentation, 199 individuals diagnosed with AHD met the inclusion criteria and were enrolled in the study. The participants were predominantly male (55.8%), with 44.2% being female. The median age was 43 years (interquartile range (IQR): 37–51 years). Employment status of the participants varied, with 38.6% being employed, 4.6% self-employed, and 52.6% unemployed. Most participants (84.9%) had AHD defined by a CD4 count of <200 cells/mm^3^, while the remainder had a WHO stage 3 condition or a CD4 percentage below 14%. Regarding treatment, 82.7% had previous exposure to antiretroviral therapy (ART) at study entry, and 17.2% were newly initiating ART (Table 1).

There was a significant difference in sex distribution between the newly and previously diagnosed groups (*p* = 0.00048). Males comprised a larger proportion of the newly diagnosed group (78.0%) compared to the previously diagnosed group (48.3%). Significant disparities were also observed in ART initiation (*p* < 0.01) with a substantially higher percentage of newly diagnosed individuals (22%) initiating ART at or after study entry compared to those that were previously diagnosed (2.34%), reflecting proactive treatment commencement among newly diagnosed individuals. There was no significant variation in CD4 counts between newly diagnosed and previously diagnosed individuals. The median CD4 counts were 87 cells/μL (IQR: 27.5–143.5) for the newly diagnosed group and 88.5 cells/μL (IQR: 49–162.8) for the previously diagnosed group (*p* = 0.26).

Among the participants, 12.1% (n = 24) had CD4 counts higher than 200 cells/μL, with a median value of 293 cells/μL. Of this group, 50% (n = 12) were determined to be WHO clinical stage 3 or 4, and 58.3% (n = 14) had a CD4 percentage lower than 14%, affirming their inclusion in the study.

### 3.2. Prevalence of Histoplasmosis and Cryptococcosis

Among the total study population, 14.6% (n = 29) were diagnosed with either histoplasmosis or cryptococcosis during the implementation of this study, which utilized rapid diagnostic assays. Histoplasmosis was the more common fungal OI, diagnosed in 11% of participants (n = 21), while 4% (n = 8) were diagnosed with cryptococcosis. 

No significant difference in the prevalence of fungal OIs was observed between participants with previous and new HIV diagnoses (Table 1). The median CD4 count among those who tested positive for a fungal OI was 49 cells/mm^3^ (IQR: 18 to 112.2). Specifically, the median CD4 count was 49 cells/mm^3^ (IQR: 21.7 to 112.2) for histoplasmosis and 49 cells/mm^3^ (15.7 to 86) for cryptococcosis (Table 2). 

Overall, 27.6% (n = 8/29) of participants diagnosed with fungal infections reported subjective risk factors for any fungal opportunistic infection (FOI) compared to 17.6% (n = 30/170) of those not diagnosed, though this difference was not statistically significant (*p* = 0.15) (Table 3).

For cryptococcosis, 38% (11/29) of participants diagnosed with FOIs reported relevant risk factors, compared to 30% (51/170) of participants without a FOI. Similarly, for histoplasmosis-specific risk factors, 24% (7/29) of those diagnosed with an OI reported specific risk factors, compared to 20% (35/170) of participants without a FOI. 

The risk of a positive diagnosis for fungal disease increases significantly as CD4 counts decrease (Table 4). No association was observed between fungal disease and demographic variables such as age, sex, respiratory symptoms, or a new HIV diagnosis. 

### 3.3. Treatment and Outcomes 

#### 3.3.1. ART Usage

Throughout the study,197 participants (98.9%) were on ART, with most initiating treatment before enrolment. Among those newly diagnosed with HIV, ART was started at a median of 14 days before study entry (IQR: 4–41). Additionally, only nine participants in this group began ART at or after study entry, with one participant initiating treatment one year after enrolment. In the previously diagnosed group, two patients started ART at the time of study entry, with the median time being 2304 days before enrolment (IQR: 872–4387)

#### 3.3.2. Antifungal Treatment 

A total of 29 patients were diagnosed with histoplasmosis and cryptococcosis, including 21 cases of histoplasmosis and 8 of cryptococcosis. Of these, 14 patients received antifungal treatment, i.e., 12 with histoplasmosis and 2 with cryptococcosis, representing 48.3% of the total population with fungal OIs (Table 5). However, 15 patients did not receive treatment, primarily due to antifungal stockouts, and in five cases, test results arrived after the patients had already passed away. Additionally, eight patients who tested negative for histoplasmosis and cryptococcosis were treated with antifungals, as most were diagnosed with oral candidiasis. Of the 22 participants (11.1%) receiving antifungal treatment, 54.5% (n = 12) were prescribed itraconazole, 54.5% (n = 12) received fluconazole, and 9.09% (n = 2) were given amphotericin B deoxycholate; both patients prescribed amphotericin B were also treated with itraconazole.

#### 3.3.3. Hospitalization and Deaths

Out of the study participants, 39 (19.6%) were hospitalized. Among them, 12 (24%) were from the newly diagnosed HIV group and 27 (18%) were from the previously diagnosed group, with no significant difference between the groups (*p* = 0.47). Of these hospitalizations, 27 occurred with a median time of four days before study entry (IQR: 3–13). Additionally, 18 individuals with fungal infections accounted for 46% of the hospitalized cases.

Seven deaths were reported within the first month of study entry, with a median time to death of 16 days (IQR: 12–21). In total, 13 deaths (6.5%) were reported, with a median time to death of 28 days (IQR: 13–81). Of these, 11 (84.6%) occurred in the previously diagnosed group, and two deaths (15.3%) occurred in the newly diagnosed group. Notably, eight deaths (61.5%) were among hospitalized participants. Men accounted for 53.8% (n = 7) of the deaths, while women represented 46.2% (n = 6).

Histoplasmosis was diagnosis in 46.2% (n = 6) of those who died, although it was not listed as the cause of death due to incomplete patient records. In all these cases, the death certificates cited the cause of death as “AIDS”. All individuals who tested positive for cryptococcus were documented as being alive.

#### 3.3.4. Kaplan–Meier Survival Analysis

**Figure 1:**
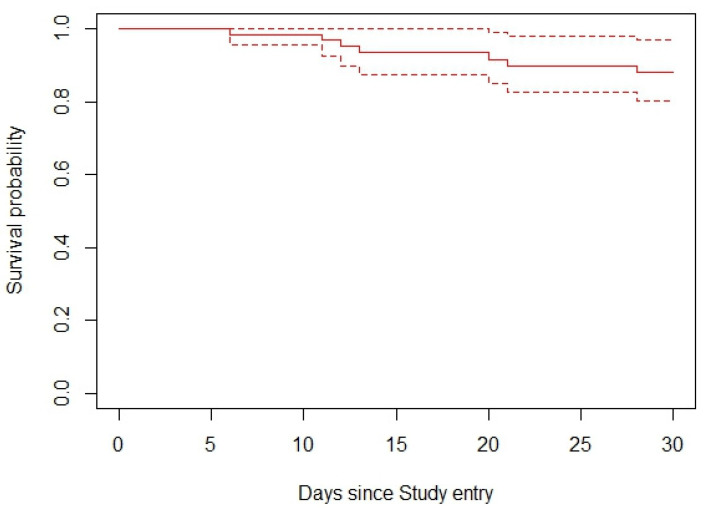
Unadjusted 30-day survival curve of participants living with AHD following study enrolment.

Kaplan–Meier survival curves were used to assess survival probabilities across different participant groups over specified durations from study entry. These analyses focused on comparing the survival probabilities among participants with AHD, distinguishing between those with and without fungal opportunistic infections (FOIs). As depicted in Figure 1, the general population’s survival probability declined progressively over the 30-day period, underscoring the ongoing risk associated with AHD.

The survival curve, represented by a solid line, was accompanied by 95% confidence intervals, shown as dashed lines. These intervals were narrow at the onset but widened over time, reflecting the increasing uncertainty in survival estimates as the number of event-free participants decreased. Despite this, the confidence intervals remained relatively narrow throughout the study period, suggesting that the survival estimates were both stable and reliable.

#### 3.3.5. Survival among Participants with and without FOIs

**Figure 2:**
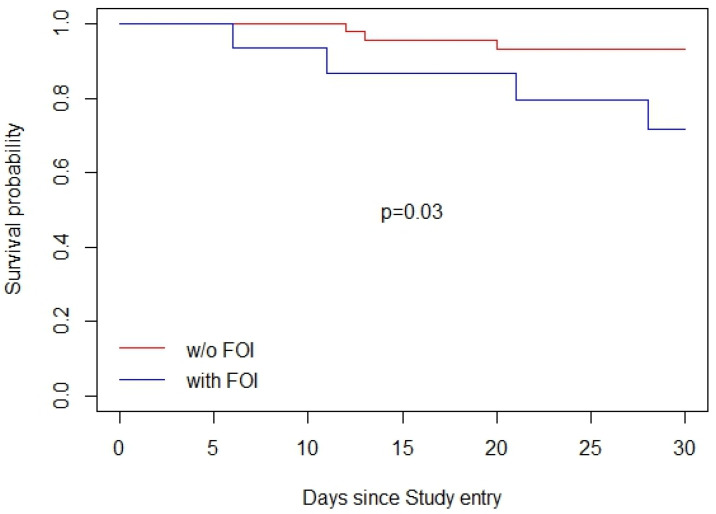
Unadjusted 30-day survival curve of participants living with AHD following study enrolment stratified by opportunistic infection diagnosis.

Two separate Kaplan–Meier analyses were conducted to assess the impact of FOIs on survival probabilities. Of the thirteen deaths reported, six occurred in the FOI group and seven in the non-FOI group, corresponding to 20. 6% and 4.1% respectively, with this difference being statistically different (*p* < 0.01). There was no significant difference in the median age between the two sub-groups (49 years, *p* = 0.83). Regarding the CD4 cell count, the FOI group had a median CD4 cell count of 48 cells/μL (IQR: 24–49), while the non-FOI group had a median of 72 cells/μL (IQR: 50–115) (*p* = 0.04). In the first analysis (Figure 2), participants with FOIs exhibited significantly lower survival probabilities over 30 days compared to those without FOIs (*p*-value of 0.03). The survival curve for participants without FOIs remained relatively stable throughout the 30-period, while those with FOIs showed a marked decline in survival probability, particularly after the first 10 days.

## 4. Discussion

### 4.1. Major Implications of Findings

Our findings highlight the critical need for the routine screening of histoplasmosis and cryptococcosis in PLHIV with AHD in Trinidad and Tobago. This recommendation aligns with the emerging global literature that emphasizes the importance of rapid and accurate diagnostic capabilities to improve patient outcomes in HIV care, particularly in regions with high prevalence rates of AHD. Notably, 12.1% of the sample included patients with a mismatch between CD4 count and CD4 percentage, which is particularly significant in the Caribbean context. These findings suggest the need to consider functional CD4 counts in individuals with conditions that can alter CD4 levels, such as sickle cell disease and HTLV-1 [25,26]. Addressing these conditions, which may mask immunosuppression, should be prioritized as a part of the screening recommendations for individuals with advanced HIV disease.

The study also emphasizes critical barriers to therapy for those diagnosed with these infections. We observed higher mortality among individuals with histoplasmosis who did not receive treatment compared to those who did. Notably, 38% of participants diagnosed with histoplasmosis showed no evidence of treatment despite clinical efforts to access medication. Similarly, 75% of participants diagnosed with cryptococcus remained untreated up to 30 days after study follow-up. The limited use of amphotericin B deoxycholate, which was prescribed in only two cases, and the lack of access to 5-flucytosine further underscore the necessity for improved access to antifungal medications. Screening, diagnosis, and timely access to affordable, lifesaving medication are essential for reducing mortality in this population.

### 4.2. Results in Comparison to Other Contexts

In regions with similar healthcare constraints, the adoption of antigen-based tests has significantly improved the detection of OIs and the clinical management of HIV/AIDS. This is particularly evident in Latin America and the Caribbean, where delays in diagnosis and treatment onset are common [18]. Our study, conducted in Trinidad and Tobago, mirrors these global findings and underscores the pivotal role of antigen-based tests in increasing diagnostic opportunities, which are crucial for timely therapeutic interventions.

Our study identified histoplasmosis in 10% and cryptococcosis in 4% of participants, paralleling findings from Trinidad and Tobago’s largest HIV treatment site, where these infections were identified in 6% and 3% of PLHIV with CD4 counts < 350 cells/mm^3^, respectively [15]. These detection rates reflect significant diagnosis capabilities compared to other regional studies. For instance, in Paraguay, the prevalence rates for histoplasmosis and cryptococcosis are approximately 12% and 10%, respectively [27]. Similarly, studies in Panama, Honduras, and Nicaragua report histoplasma antigen positivity in 20% of participants and cryptococcus antigen positivity in 11% participants [9]. These findings further highlight the importance of antigen-based testing in addressing the burden of fungal infections in HIV care across the region.

### 4.3. System Implications

The integration of antigen-based tests into the national HIV/AIDS program has far reaching implications for healthcare delivery, suggesting a shift towards more streamlined and efficient diagnostic processes that could be extended to other infectious diseases [28]. Our study demonstrates a heightened risk of fungal OIs among PLHIV with CD4 cell counts below 200 cells/mm^3^. This finding aligns with the WHO’s recommendation to strengthen health systems’ diagnostic capacity to improve global treatment outcomes for infectious diseases [29]. We advocate for making antigen-based diagnostic assays for FOIs a standard screening tool for all PLHIV with AHD presenting with suspicious symptoms of AHD regardless of the CD4 count.

Despite efforts to secure antifungal medication and notify clinical teams, access to treatment remained a significant challenge for the cohort. A follow-up system-strengthening initiative is crucial to enhance both diagnostic capabilities and treatment access.

Additional considerations include monitoring both CD4 counts and CD4 percentages to determine optimal screening triggers for OIs in individuals with AHD. A major limitation observed was the restricted access to antifungal treatment for those who tested HIV-positive. Expanding fungal infection screening will enable patients with advanced diseases to access therapy sooner, particularly given the cost and availability disparities between itraconazole, fluconazole, and amphotericin B. Timely access is essential as it directly influences the treatment outcome and survival rates of PLHIV with AHD.

### 4.4. Future Research

Future studies should prioritize on longitudinal assessments to better understand the long-term effects on survival rates and quality of life among PLHIV as antigen tests are scaled up at a national level, particularly in countries with limited access to diagnostics and medicines. Additionally, there is a need to explore the clinical presentation of fungal infections within the local cohorts and the factors affecting access to treatment. Evaluating the cost-effectiveness of widespread antigen test implementation in resource-limited settings will be essential to support its integration into national health programs [30]. Further research should also investigate the scalability of these diagnostic tools and their adaptation for detecting other prevalent infectious diseases, which could substantially enhance public health strategies.

### 4.5. Limitations

Despite the robust design of our study, potential sources of error stem from the inherent limitations of antigen-based tests, including their sensitivity and specificity in field conditions. Additionally, during the study period, diagnostic tools to support gold-standard culture testing were unavailable, preventing the independent validation of test results. The study’s reliance on single-time-point data collection may also limit the ability to capture disease progression dynamics or access the long-term impact of diagnostic tests on health outcomes.

### 4.6. Conclusions

The implementation of antigen-based tests for histoplasmosis and cryptococcosis represents a transformative advancement in managing HIV-related opportunistic infections, offering critical insights into the immediate and tangible benefits that these tools offer in clinical practice. Our study not only reinforces the continued adoption of these diagnostic technologies but also calls for a broader reconsideration of their potential as infectious disease diagnostics across diverse healthcare settings.

## Figures and Tables

**Table 1:** Sociodemographic and clinical characteristics of included patients with previous and new HIV diagnoses.

Characteristics	New HIV Diagnosis 50 (25.1%)		Previous HIV Diagnosis 149 (74.9%)		Total 199 (100%)		*p*-Value, x^2^, Kruskal
	n	%	n	%	n	%	
**Sex**							
Female	11	22.0	77	51.7	88	44.2	0.00048
Male	39	78.0	72	48.3	111	55.8	
**Age**							
Median (IQR)	40.5	(33.2–49.7)	44.0	(37.0–52.0)	43.00	(37.0–51.0)	0.338
**Employment status**							0.028
Employed	26	53.1	49	33.8	75	38.6	
Self-employed	4	8.2	5	3.4	9	4.6	
Retired	1	2.0	7	4.8	8	4.1	
Unemployed	18	36.7	84	57.9	102	52.6	
**ART initiation**							<0.01
Before study entry	38	76.0	146	97.9	184	92.4	
At/after study entry	11	22.0	2	2.34	13	6.5	
Unknown	1	2.0	1	0.67	2	1.0	
Newly initiated ART *							<0.01
No	18	36.7	145	97.9	163	82.7	
Yes	31	63.3	3	2.0	34	17.2	
**CD4 <= 200**							0.129
No	3	6.0	21	14.1	24	12.1	
Yes	44	88.0	125	83.9	169	84.9	
Unknown	3	6.0	3	2.0	6	3.0	
**Median CD4 value**	87	(27.5–143.5)	88.5	(49.0–162.8)	87.0	(47.0–160.0)	0.26
**Type of patient**							0.49
Outpatient	41	82.0	130	87.2	171	85.9	
Inpatient	9	18.0	19	12.7	28	14.1	
Any fungal OI **	5	10.0	24	16.1	29	14.6	0.40
Histoplasmosis	4	8.0	17	11.4	21	10.5	0.67
Cryptococcosis	1	2.0	7	4.7	8	4.0	

* Defined as initiated ART within 30 days before and after study entry. ** Determined in the study, and prior fungal infection was an exclusion criterion.

**Table 2:** Distribution of patients and CD4 counts by fungal opportunistic infections.

	Total Positive	Total Histoplasmosis	Total Cryptococcosis
Number of patients (%)	29 (14.6%)	21 (10.5%)	8 (4.0%)
Median CD4 count (cells/mm^3^) (IQR)	49 (18–112.2)	49 (21.7–112.2)	49 (15.7–86)
Missing CD4 count (%)	3	3	0

Notes: percentages relate to the total number of patients, n = 199.

**Table 3:** Self-reported risk factors for acquisition of fungal opportunistic infections by populations diagnosed with or without fungal opportunistic infections *.

Risk Factor	Total Population (N = 199)	Population with FOIs (N = 29)	Population without FOIs (N = 170)
**Any risk factor for cryptococcosis**	62 (31.1%)	11 (37.9%)	51 (30%)
Exposure to bird or pigeon excreta	38 (19.1%)	8 (27.5%)	30 (17.6%)
Involved in ground/soil removal	32 (16.2%)	3 (10.3%)	29 (17.1%)
Involved in demolition work	17 (8.6%)	2 (6.9%)	15 (8.8%)
**Any risk factor for histoplasmosis**	42 (20.0%)	7 (24.1%)	35 (20%)
Exposure to bat guano/droppings	26 (13.0%)	5 (17.2%)	21 (12.3%)
Involved in ground/soil removal	32 (16.2%)	3 (10.3%)	29 (12.3%)
Involved in demolition work	17 (8.6%)	2 (6.9%)	15 (8.8%)
Visited caves	3 (1.5%)	0 (0%)	3 (1.5%)
Previous trips/outdoor activities	16 (8.1%)	4 (13.8%)	12 (7.1%)
**Any risk factor for fungal OI**	38 (19.6%)	8 (27.6%)	30 (18.1%)

* None of the risk factors was associated with FOI vs. non-FOI.

**Table 4:** Logistic models for fungal opportunistic infections and associated factors.

Variable	Any FOI		Any FOI Imputed *	
	aOR	95%CI	aOR	95% CI
**Sex** Female vs. male	1.02	0.40–2.58	0.85	0.35–2.02
**Age** 30 years 40 years 50 years	1 1.27 1.68	0.48–3.35 0.21–13.3	1 1.06 1.05	0.44–2.54 0.33–3.26
**Any respiratory clinical manifestation**	1.71	0.68–4.28	1.99	0.86–4.60
**New HIV diagnosis status**	0.45	0.13–1.50	0.44	0.15–1.30
**CD4 Count**				
50 cells/mm^3^	2.88	1.26–6.62	3.05	1.33–6.97
100 cells/mm^3^	1.68	0.89–3.16	1.72	0.90–3.29
200 cells/mm^3^	1		1	
**Risk factors for fungal OIs**	1.79	0.64–4.96	1.09	0.46–2.58

* Multiple imputations were used for the 6 cases without a CD4 count; aOR = adjusted odds ratio.

**Table 5:** Antifungal therapy for people with advanced HIV disease based on fungal opportunistic infections.

Antifungal Therapy	Among Patients with Positive Histoplasmosis Test (n = 12/21)	Among Patients with Positive Cryptococcosis Test (n = 2/8)	No Histoplasmosis or Cryptococcosis Confirmed (n = 8)
Itraconazole	11	0	1
Fluconazole	2	2	8
Amphotericin B	2 *	0	1

Note: Antifungal therapy results are not mutually exclusive. One participant with histoplasmosis received all three drugs, and one received itraconazole and amphotericin. From participants with no FOI, one received all three drugs. * Combined with itraconazole.

## Data Availability

The original contributions presented in the study are included in the article, further inquiries can be directed to the corresponding author.
