# Correction: Sebro et al. Implementation of Antigen-Based Diagnostic Assays for Detection of Histoplasmosis and Cryptococcosis among Patients with Advanced HIV in Trinidad and Tobago: A Cross-Sectional Study. *J. Fungi* 2024, *10*, 695

**DOI:** 10.3390/jof10120806

**Published:** 2024-11-21

**Authors:** Ayanna Sebro, Jonathan Edwards, Omar Sued, Leon-Omari Lavia, Tricia Elder, Robert Jeffrey Edwards, Patrick Eberechi Akpaka, Nadia Ram-Bhola, Roanna Morton-Williams Bynoe, Yanink Caro-Vega, Isshad John, Freddy Perez

**Affiliations:** 1 National AIDS Coordinating Committee, Office of the Prime Minister, Port of Spain 190126, Trinidad and Tobago; ayanna.sebro@gov.tt; 2 HIV/AIDS Coordinating Unit Ministry of Health, Port of Spain 101002, Trinidad and Tobago; jonathan.r.a.edwards@gmail.com (J.E.); omari.lavia@health.gov.tt (L.-O.L.); tricia.elder@health.gov.tt (T.E.); nadia.ram-bhola@health.gov.tt (N.R.-B.); roanna.m-w-bynoe@health.gov.tt (R.M.-W.B.); isshad.john@health.gov.tt (I.J.); 3 Department of Communicable Diseases Prevention, Control, and Elimination, Pan American Health Organization, Washington, DC 20037, USA; suedoma@paho.org; 4 Medical Research Foundation, 7 Queens Park East, Port of Spain 101002, Trinidad and Tobago; jeffreye2000@gmail.com; 5 Department of Paraclinical Sciences, The University of the West Indies, St. Augustine 330912, Trinidad and Tobago; eberechipatrick.akpaka@uwi.edu; 6 Departamento de Infectología, Instituto Nacional de Ciencias Médicas y Nutrición Salvador Zubirán, Tlalpan, México City 14080, Mexico; yanink.caro@infecto.mx; 7 Department of Community Health, Federal University of Health Sciences of Porto Alegre (UFCSPA), Porto Alegre 90050-170, RS, Brazil

## Authorship Correction

In the original publication [1], there was an oversight in recognizing the contributions of Robert Jeffrey Edwards and Patrick Eberechi Akpaka, who were incorrectly included in the acknowledgment section of the manuscript. The list of authors and their affiliations should now read as follows:

Ayanna Sebro ^1^, Jonathan Edwards ^2^, Omar Sued ^3,†^, Leon-Omari Lavia ^2^, Tricia Elder ^2^, Robert Jeffrey Edwards ^4^, Patrick Eberechi Akpaka ^5^, Nadia Ram-Bhola ^2^, Roanna Morton-Williams Bynoe ^2^, Yanink Caro-Vega ^6^, Isshad John ^2^ and Freddy Perez ^3,7,^*^,†^

## Affiliation Correction

With the inclusion of the two authors (Robert Jeffrey Edwards and Patrick Eberechi Akpaka), their affiliations need to be included. 

Additionally, in the original publication [1], a legal statement for the two authors affiliated with the Pan American Health Organization (Omar Sued and Freddy Perez) was missing, some affiliations also need to be adjusted and improved. This now needs to read as follows:

1 National AIDS Coordinating Committee, Office of the Prime Minister, Port of Spain 190126, Trinidad and Tobago; ayanna.sebro@gov.tt
2 HIV/AIDS Coordinating Unit Ministry of Health, Port of Spain 101002, Trinidad and Tobago; jonathan.r.a.edwards@gmail.com (J.E.); omari.lavia@health.gov.tt (L.-O.L.); tricia.elder@health.gov.tt (T.E.); nadia.ram-bhola@health.gov.tt (N.R.-B.); roanna.m-w-bynoe@health.gov.tt (R.M.-W.B.); isshad.john@health.gov.tt (I.J.)
3 Department of Communicable Diseases Prevention, Control, and Elimination, Pan American Health Organization, Washington, DC 20037, USA; suedoma@paho.org
4 Medical Research Foundation, 7 Queens Park East, Port of Spain 101002, Trinidad and Tobago; jeffreye2000@gmail.com
5 Department of Paraclinical Sciences, The University of the West Indies, St. Augustine 330912, Trinidad and Tobago; eberechipatrick.akpaka@uwi.edu
6 Departamento de Infectología, Instituto Nacional de Ciencias Médicas y Nutrición Salvador Zubirán, Tlalpan, México City 14080, Mexico; yanink.caro@infecto.mx
7 Department of Community Health, Federal University of Health Sciences of Porto Alegre (UFCSPA), Porto Alegre 90050-170, RS, Brazil

* Correspondence: perezf@paho.org; Tel.: +1-202-2576715
^†^  The author is a staff member of the Pan American Health Organization. The author alone is responsible for the views expressed in this publication, and they do not necessarily represent the decisions or policies of the Pan American Health Organization.

## Author Contributions Correction

With the inclusion of the two authors in the authorship, the author contributions should now read as follows:

**Author Contributions:** Conceptualization, F.P., O.S., A.S. and I.J.; methodology, F.P. and O.S.; formal analysis, L.-O.L. and Y.C.-V.; resources, F.P.; writing—original draft preparation, A.S., J.E., F.P., O.S. and Y.C.-V.; writing—review and editing, A.S., J.E., O.S., L.-O.L., T.E., R.J.E., P.E.A., N.R.-B., R.M.-W.B., Y.C.-V., I.J. and F.P. All authors have read and agreed to the published version of the manuscript.

## Acknowledgement Correction

In the original publication [1], we acknowledged Robert Jeffrey Edwards and Patrick Eberechi Akpaka for their support in protocol development, oversight and comments to draft versions of the manuscript. This statement should be removed as they have now been included in the authorship. This section should now read as follows:

**Acknowledgments:** We would like to acknowledge the laboratory and clinical teams across all study sites, including the study team coordinators and surveillance nurses for their continuous support. Special thanks are due to Diego Caceres (IMMY Diagnostics), Alex Jordan (Centers for Disease Control and Prevention) and Narda Medina (Asociación de Salud Integral, Guatemala) for their invaluable contributions during the implementation phase of the study. Additionally, we are grateful to the HIV/AIDS Coordinating Unit (HACU), Ministry of Health, Trinidad and Tobago; the National AIDS Coordinating Committee Secretariat (NACC) of Trinidad and Tobago; the Department of Para-Clinical Sciences of the University of the West Indies, St Augustine Campus; the Trinidad Public Health Laboratory; the Thoracic Medical Team of the Ministry of Health, Trinidad and Tobago, for their continuous support throughout this study. Finally, we express our gratitude to all participants and their families.

The authors state that the scientific conclusions are unaffected. This correction was approved by the Academic Editor. The original publication has also been updated.
